# Effect of a facility-based multifaceted intervention on the quality of obstetrical care: a cluster randomized controlled trial in Mali and Senegal

**DOI:** 10.1186/1471-2393-13-24

**Published:** 2013-01-25

**Authors:** Catherine M Pirkle, Alexandre Dumont, Mamadou Traoré, Maria-Victoria Zunzunegui

**Affiliations:** 1 Research Centre of the University Hospital Centre of Quebec (CHUQ), 2875 boulevard Laurier, Édifice Delta II, Bureau 600, 6e étage, Québec, Québec, GIV 2M2, Canada; 2 Department of Social and Preventive Medicine, University of Montreal, Montreal, Canada; 3 Research Centres of the University of Montreal Hospital Complex, Hôtel Dieu, Pavillon Masson, Montréal, Québec, Canada; 4 Research Institute for Development, Université Paris Descartes, Sorbonne Paris Cité, UMR 216, Paris, France; 5 URFOSAME, District health centre of Commun V, Bamako, Mali

**Keywords:** Maternal death review, Criterion based clinical audit, Quality of care, Obstetrics, West Africa

## Abstract

**Background:**

Maternal mortality in referral hospitals in Mali and Senegal surpasses 1% of obstetrical admissions. Poor quality obstetrical care contributes to high maternal mortality; however, poor care is often linked to insufficient hospital resources. One promising method to improve obstetrical care is maternal death review. With a cluster randomized trial, we assessed whether an intervention, based on maternal death review, could improve obstetrical quality of care.

**Methods:**

The trial began with a pre-intervention year (2007), followed by two years of intervention activities and a post-intervention year. We measured obstetrical quality of care in the post-intervention year using a criterion-based clinical audit (CBCA). We collected data from 32 of the 46 trial hospitals (16 in each trial arm) and included 658 patients admitted to the maternity unit with a trial of labour. The CBCA questionnaire measured 5 dimensions of care- patient history, clinical examination, laboratory examination, delivery care and postpartum monitoring. We used adjusted mixed models to evaluate differences in CBCA scores by trial arms and examined how levels of hospital human and material resources affect quality of care differences associated with the intervention.

**Results:**

For all women, the mean percentage of care criteria met was 66.3 (SD 13.5). There were significantly greater mean CBCA scores in women treated at intervention hospitals (68.2) compared to control hospitals (64.5). After adjustment, women treated at intervention sites had 5 points’ greater scores than those at control sites. This difference was mostly attributable to greater clinical examination and post-partum monitoring scores. The association between the intervention and quality of care was the same, irrespective of the level of resources available to a hospital; however, as resources increased, so did quality of care scores in both arms of the trial.

**Trial registration:**

The QUARITE trial is registered on the Current Controlled Trials website under ISRCTN46950658

## Background

Each day, 800 women worldwide die during pregnancy or of childbirth-related causes. Most maternal deaths occur in sub-Saharan Africa [1]. Globally, it is recognized that significant inroads in maternal mortality cannot be made without dramatically increasing access to emergency obstetrical care (EmOC) [2]. Hospitals capable of basic EmOC should provide parental antibiotics, anticonvulsants, and oxytocics, as well as be able to manually remove the placenta and retained uterine products, and perform assisted deliveries. Comprehensive EmOC sites also perform caesarean sections and blood transfusions [3]. Yet, in many places, the numbers of maternal deaths occurring in health facilities providing EmOC are unacceptably high. In Mali and Senegal, over one percent of women die giving birth in referral hospitals across the region, in places where comprehensive EmOC is available [4]. Such high case fatality suggests that poor medical practice may contribute to maternal mortality, along with other factors such as long travel times and late recognition of obstetrical complications.

While elevated facility maternal mortality indirectly suggests deficiencies in quality of care, little work in the region has directly measured the problem. Anthropologists working in five West African capital cities have documented shocking departures from quality obstetrical care. These ranged from gross neglect to physical violence against patients [5]. Additionally, limited quantitative evidence has indicated that over 70% of maternal deaths in the region could be avoided with better quality care [6]. While sobering, this work does not quantify the amplitude of the quality problem nor give health professionals concrete suggestions on how to improve obstetrical practice.

Recently, there has been substantial interest in employing maternal death review (MDR) to improve obstetrical quality of care and reduce maternal mortality [7-10]. MDR are audits of maternal deaths in which all healthcare staff involved in the management of a particular patient discuss the circumstances of the death and how care could have been improved. During the audit, recommendations to improve future patient care are made [7]. In Senegal, a pilot study using MDR showed a 50% reduction in maternal mortality [11]. MDR are believed effective at improving obstetrical care because they draw hospital staff attention to the problem of maternal mortality, highlight quality gaps, and emphasize finding solutions that are realistic in a given setting. Overall, they are believed to increase provider accountability.

In September, 2007, the QUARITE cluster randomized trial was initiated to assess the effect of a multifaceted intervention, the ALARM International Program, on reducing facility maternal mortality [4]. The hallmark of the intervention is MDR. Other aspects of the intervention include workshops on obstetrical best practices and periodic visits to ALARM sites by international experts. It is believed that the workshops and international visits facilitate MDR by providing health care professionals with the knowledge and confidence to make quality improvement suggestions during the reviews. In all, the ALARM program targets heath professionals in maternity units and is believed to reduce maternal mortality *by improving quality of care,* through better obstetrical practice. For the trial, we randomly allocated the ALARM intervention to referral hospitals in the region. To avoid contamination between health professionals, and because the intervention targets teams of professionals, a cluster design was deemed more appropriate than an individual randomized controlled trial.

## Methods

The QUARITE trial began in September 2007 and was completed in December 2011. It consisted of a one-year pre-intervention period, a two-year intervention period, and a final year post-intervention period. A detailed description of the trial design and objectives has been described elsewhere [4]. The trial received ethics committee approval from Sainte Justine Hospital in Montreal (ref. 2425), the Ministry of Health and Preventive Medicine in Senegal (ref. 0869), and the National Ethics Committee for Health and Life Sciences in Mali (ref. 034/MS-SG-CNESS). Informed consent was obtained from each hospital included in the trial.

### Setting

The QUARITE trial took place in referral hospitals in Mali in Senegal. Referral hospitals are evacuation sites for complicated deliveries and in theory, should have sufficient infrastructure to provide comprehensive EmOC including caesarean section and blood transfusion [3]. In both countries, there are sufficient numbers of comprehensive EmOC facilities for the population, though rural areas are underserved [12]. In Mali, approximately 45% of women give birth in a basic or comprehensive health facility and of those whose births were assisted by a health professional, 2% received a caesarean section [13]. In Senegal, the respective proportions are 62% and 3% [14].

### Participants

As this is a cluster design, eligibility criteria apply to both the hospital and patient levels of analysis. To be eligible for the QUARITE trial, the referral hospital needed to have comprehensive EmOC capacity (i.e. a functioning surgical theatre and blood transfusion capacity) and at least 800 births per year. We focused on high volume hospitals in order to have enough maternal deaths (main trial outcome) and because the health system is organized in a pyramidal fashion. That is, lower order health centres refer cases of complication up to higher level, larger facilities that, in theory, should be able to able to treat life-threatening obstetrical complications. QUARITE’s eligibility criteria have been described in detail elsewhere, and a total of 46 of the 49 eligible referral hospitals were included in the trial [4]. For the present study and analyses, all women admitted to hospital with a trial of labour and a foetus of at least 500gms were eligible for this sub-study. Women admitted for elective caesarean section were not included, because they did not have a trial of labour. Elective caesarean section accounted for two percent of all birth in the trial [15].

We sampled the charts of two populations of women. Providers were not aware of which charts would be sampled until the day of data collection which was after all care had been provided to the obstetrical patient. The first sample, called the consecutive sample, included the last 20–25 births having occurred at a hospital as of September 30, 2010. It was a random snap shot of the population of women attending a given hospital on an arbitrary date and included some of the women in the second sample. The second sample, called the complicated sample, included only women with severe pre-eclampsia and/or eclampsia and post-partum haemorrhage (defined below). We only looked at pre-eclampsia and haemorrhage, because these were the two most frequent and lethal complications of women treated in trial hospitals, according to data collected during the baseline year of QUARITE. Women in the complicated sample were selected within a three-month sampling frame: July-September, 2010 in Senegal and September-November, 2010 in Mali. We aimed for 10–15 complications per hospital, starting in September and working backwards in Senegal (and forwards in Mali) until the desired sample size was achieved or the sampling frame was exhausted. The dates of the sampling frames differ slightly because of when data was collected and the availability of records for audit (Mali was sampled after Senegal).

To locate cases of severe (pre-) eclampsia/ eclampsia and post-partum haemorrhage, we used three data sources: the delivery register, the patient medical chart, and the QUARITE trial data sheet (records basic information on all women giving birth in trial hospitals, including diagnoses of obstetrical complication). Prior to QUARITE trial commencement, staff at both intervention and control sites received training on the diagnosis of obstetrical complications according to international guidelines. This was done to ensure consistency in the definitions of complication used across sites.

For this sub-study, we assessed the care of patients with severe pre-eclampsia/ eclampsia and post-partum haemorrhage. To locate cases of severe pre-eclampsia or eclampsia, we searched the three forms of medical records described above. We looked for a diagnosis of pre-eclampsia, toxaemia, or eclampsia, as recorded by a healthcare professional (usually a midwife or doctor). Since many pre-eclampsia patients had mild to moderate pre-eclampsia, we also looked for the following indications of *severe* pre-eclampsia: mention of severity, convulsions, albumin laboratory scores of +++, and vomiting. For post-partum haemorrhage, in addition to an express diagnosis of the complication, we looked in depth at patients with antenatal haemorrhage (placenta praevia, abruptio-placentae) continuing into the post-partum period. We looked for indicators of postpartum haemorrhage such as: an important postpartum drop in blood pressure and/or post-partum blood pressure below 90/50 [16]; a postpartum drop in haemoglobin levels [17], retained placental fragments or incomplete expulsion of the placenta, mention of uterine atony, intravenous post-partum administration of oxytocin or post-partum blood transfusion. We did not include blood loss because this was not recorded in the patient medical records and because physiological change may be more appropriate in this context, as there is a high prevalence of anaemia [18]. The case notes for each obstetrical complication were verified by an obstetrician gynaecologist (AD) in order to reduce the inclusion of false positives.

### Intervention

The ALARM International Program was developed by the Society of Obstetricians and Gynaecologists of Canada. It combines clinically-oriented and evidence-based outreach visits with facility-based MDR. Specifically, the intervention included the following activities: 1) A six-day workshop to train and certify opinion leaders (one physician and midwife from each intervention site) in EmOC best practices, audit techniques, and sexual and reproductive rights; 2) the creation of a multidisciplinary audit committee (physicians, midwives, nurses, and administrators) at each site; 3) commencement of a once-monthly audit cycle according to WHO guidelines [7]; 4) the training of qualified staff in obstetrical best practices with 4–8 training sessions during the intervention period organized by local opinion leaders and external facilitators; 5) Educational outreach every three months by external facilitators including a national opinion leader and an ALARM international coordinator to support local opinion leaders in their activities and; 6) Recertification of local opinion leaders a year after initial certification with an accelerated training workshop. A more detailed description of the intervention has been published elsewhere [4]. The control group did not receive any intervention by the research team after the baseline year. In other words, normal practice continued at the control sites including infrequent training courses (mostly related to HIV care) for obstetricians and midwives and some oversight by regional and national governments regarding the numbers of maternal deaths and prevention of mother to child transmission of HIV. It should be noted that these same activities were also carried out at ALARM sites. The ALARM program was implemented in addition to existing activities in the region.

### Objectives

The primary objective of the trial was to reduce facility maternal mortality (*results forthcoming*). Secondary objectives of the trial included: 1) reducing stillbirth and neonatal mortality; 2) reducing severe maternal morbidity; 3) improving quality of care and 4) increasing health professional job satisfaction [4]. In this paper, we address the secondary objective of improving quality of care, as it is the mechanism by which the ALARM intervention is believed to reduce maternal mortality. In other words, we did not expect significant reductions in facility maternal mortality without first seeing improvements in obstetrical care.

Here, we evaluate the effect of ALARM on obstetrical quality of care during labour, delivery and the immediate post-partum period. It is during this period that most maternal deaths occur [19]. We assess whether quality of care at the patient level differs according to trial arms (ALARM versus control sites) and whether certain aspects of the care process have been differentially affected by the intervention.

### Outcomes

The primary outcome of this study was patient intrapartum quality of care, as measured with chart abstraction (see below). Secondary outcomes for this study were: scores for different dimensions of quality, quality scores for severe pre-eclampsia and eclampsia, and quality scores for postpartum haemorrhage.

Quality of care was assessed with a form of chart abstraction known as criterion-based clinical audit (CBCA) [7]. When using CBCA, standardized criteria for evaluating good quality of care are predetermined, usually with medical literature and expert opinion, and then compared against data extracted from medical records to evaluate whether or not a minimal standard of care has been met [5,14]. CBCA is formatted to look like a checklist of pre-specified standards of good care. For example, the auditor may “check-off” if a patient’s blood pressure, cardiac frequency, and temperature were taken. For each affirmative response related to an expected standard of care, a point is given. The points are tallied up and divided by the total possible in order to assign a quality of care score to each patient.

The CBCA that we used has been described extensively elsewhere [20] and is both a valid and reliable tool for measuring obstetrical quality of care. Prior to conducting the audit, we piloted the questionnaire on 185 medical charts to assess inter-rater reliability and to maximize content validity. Additional analyses of the CBCA instrument showed that low scores (less than 70% criterion attainment) predict perinatal mortality; this strongly indicates construct validity [20]. Additionally, because of concerns that missing patient obstetrical charts might lead to selection bias [21], we systematically recorded the numbers of missing records and patient characteristics (patient age; vaginal birth versus caesarean section) based on data collected from the delivery register. We assessed if there was an association between patient characteristics and missing obstetrical records and found no association. This suggests that records were missing at random, as patient characteristics for retrieved and non-retrieved records were similar [20].

The CBCA contains 26 unweighted criteria that measure 5 dimensions of care: patient history, clinical examination, laboratory examinations, labour management (partograph), delivery care and postpartum monitoring. These sections apply to all women sampled. A quality of care score was attributed to each woman based on the number criteria met. For example, if 20 criteria were attained during the audit of a given medical record, then the score for that record would be 20/26 or 76.9%. In accordance with previous studies [7], we also defined a binary outcome to assess quality of care as follows: good care defined as greater than 70% criterion attainment versus moderate to poor care defined as 70% or less attainment [20]. Finally, two sections of the questionnaire applied only to women with severe pre-eclampsia/eclampsia and postpartum haemorrhage. These were scored separately (denominator of 7 for each). Table 1 shows the criteria included for each section.

**Table 1:** Criteria included in the CBCA to measure obstetrical quality of care

**Dimension**	**Criteria**
History taking	· Condition of the mother at arrival
	· Number of prenatal examinations
	· Age
	· Gravidity
	· Parity
Clinical examination at admission	· Uterine height
	· Pulse
	· Blood Pressure
	· Temperature
	· Foetal presentation
	· Foetal heart beat
	· Membranes/amniotic fluid
	· Cervical dilation
Laboratory analyses	· Blood type
	· Rhesus factor
	· HIV test
	· Syphilis test
Monitoring during birth	· Name of person who assisted the birth
	· Qualification of the birth attendant
	· Time of placental expulsion
	· Oxytocin given
	· Time of birth given
Postpartum monitoring	· Follow-up examination
	· Exit examination
	· Date of exit
	· Condition of the infant at birth
Severe pre-eclampsia and eclampsia (*specific to women with this diagnosis only*)	· Anticonvulsant administered
	· Blood pressure recorded every four hours after birth
	· Urinary output measured at least once in 24 hours
	· Test of bleeding time
	· Test of coagulation rate
	· Platelet count
	· Albumin test
Post-partum haemorrhage (*specific to women with this diagnosis only*)	· Pulse and blood pressure every 15 minutes for 2 hours after diagnosis
	· Injection of oxytocin or egometrine
	· Intravenous oxytocin perfusion
	· Placenta expulsed
	· Test of bleeding time
	· Test of coagulation rate
	· Platelet count

### Sample size

We used Hayes and Moulton’s (2009) formula for cluster randomized trials with a quantitative endpoint [22]. Our endpoint was mean patient CBCA score. Previous work in low- and middle- income countries has shown post-audit improvements in quality of care scores of 15-35% [21]. We conservatively estimated that mean patient CBCA scores at ALARM hospitals would be 15% greater than at control sites. We assumed that standard deviations around the CBCA score would be the same in both groups and estimated them to be 0.15. We expected a high degree of within cluster correlation in CBCA scores, because facility institutional culture would likely lead similar obstetrical care and recording practices. We thus used a highly conservative intra-class correlation coefficient of 0.50. Given that we planned to sample 20 medical charts per hospital for the consecutive sample, 9 hospitals per trial arm would be necessary to detect a significant difference between intervention and control groups, with 80% power and an alpha of 0.05. For the subsamples of post-partum haemorrhage and severe pre-eclampsia and eclampsia, which used only 10 medical charts per site, we would need 10 hospitals per trial arm to detect a significant difference between arms.

### Randomization

Centres were included on the basis of formal, informed consent on the part of the hospital director and the person in charge of maternity services. After a one-year pre-intervention data collection phase, each hospital was randomly assigned (August 2008) to either an intervention group, in which ALARM was implemented, or a control group.

The participating hospitals were stratified into six strata corresponding to the combination of two countries (Mali and Senegal) and three hospital types: hospitals in the capital, regional hospitals, and district hospitals outside the capital. We attempted to ensure optimal balance between the hospitals assigned to the intervention and the control groups in terms of their number and size (number of deliveries per year). Therefore, within each stratum, we first ranked the hospitals with respect to size, and then used blocked randomization, with each block of size two, containing two hospitals with adjacent ranks, i.e., of similar size. All participating hospitals were randomized simultaneously, after their list was provided, which eliminated any risk of allocation bias.

### Blinding

Patients attending the study facilities were blinded to group assignment. For obvious reasons, those administering the intervention and health professionals implementing the intervention were not blinded.

### Statistical methods

All statistical analyses were conducted in SPSS 17.0. We used t-tests to assess if there were statistically significant differences in CBCA scores according to arms of the QUARITE trial (ALARM versus control). We used mixed models, which take into account clustering by hospital, to examine predictors of CBCA score. Because of the limited number of clusters in most CRTs, it is not unusual to find imbalances in covariates between trial arms [22]. Thus, at the cluster level, we adjusted for country, material and human resources available for obstetric care (see below), and capital versus regional location. At the patient level, we assessed for confounding by age, parity and the number of prenatal consultations. We entered each patient variable one at a time to evaluate changes in the coefficient for ALARM. Changes of greater than 10% were considered potential confounding.

For each site, we collected information on material and human resources allowing us to score the structural capacity of each centre. This score, called the Complexity Index Score, was derived from an instrument used in the WHO Global Survey on Maternal and Perinatal Health [23]. It is comprised of eight categories describing: 1) basic services (e.g. water, electricity, etc.), 2) screening tests (proteinuria, urine culture, etc.), 3) basic emergency obstetrical resources (antibiotics, hysterectomy, transfusion, etc.), 4) intrapartum care (partograph use, skills in forceps, etc.), 5) general medical services (e.g. medical laboratory, sterilization equipment, etc.), 6) anaesthesiology resources (anaesthesiologist present 24h, equipment for general anaesthesiology, etc.) 7) human resources (nurse, midwife, etc.) and, 8) academic resources and clinical protocols (medical library, internet, etc.). We used an Africa-specific grading scheme for the Index proposed by Shah [24]. A list of services under each of the categories described above is classified as essential, comprehensive, or advanced. Each service classified as essential receives one point, each comprehensive service receives two points, and each advanced service gets 3 points. Points for the Complexity Index were summed up for each hospital. Scores can vary from 0 to 100. For the Complexity index score, we centred the values around the mean to make the results more easily interpretable.

Because the Malian and Senegalese health systems are organized differently, we also assessed for effect modification by country. Similarly, we assessed for effect modification by Complexity Index Score, hypothesizing that ALARM would be most effective at sites with greater resources.

## Results

### Cluster (hospital) flow

**Figure 1:**
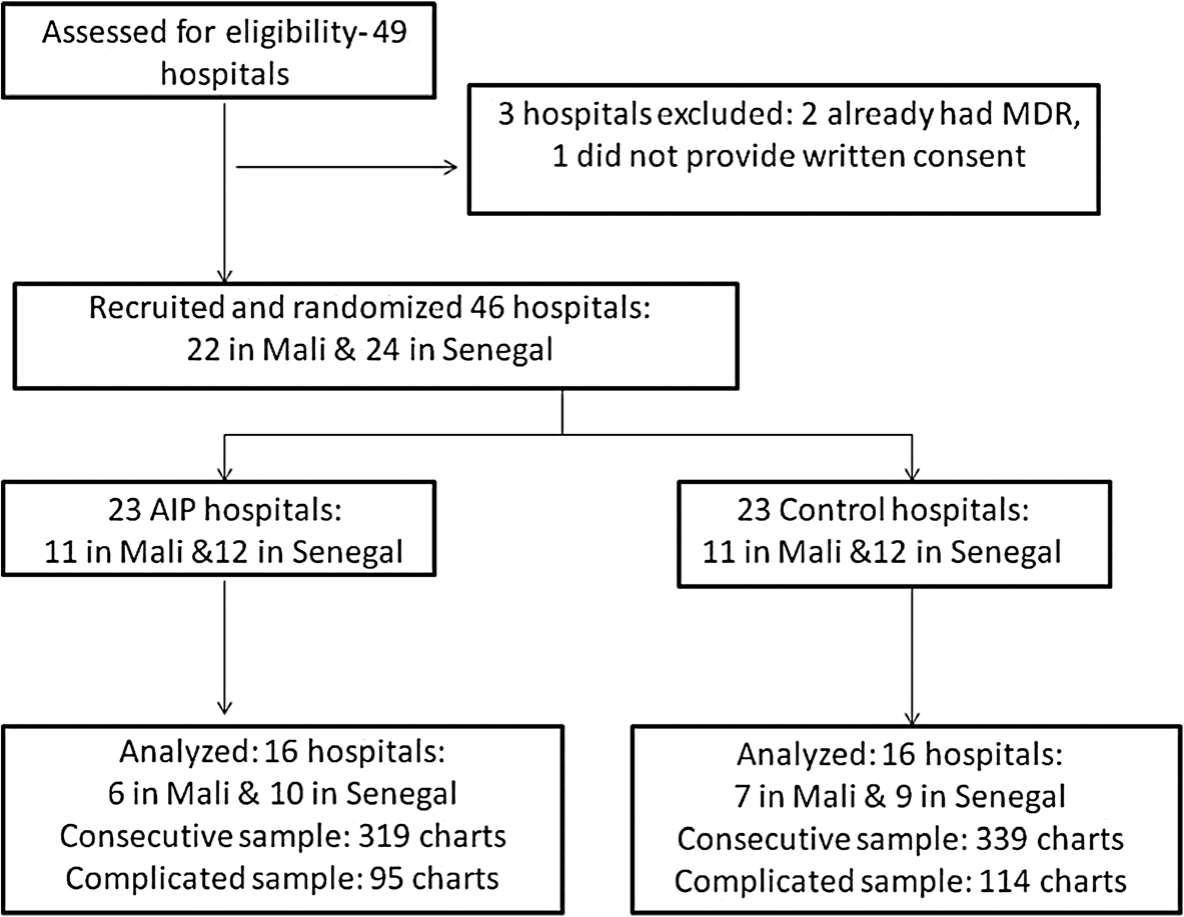
Flow diagram of clusters and medical charts included in the study.

Figure 1 shows the numbers of clusters followed through each stage of the study. Because of logistical and financial constraints, *for this study* on quality of care, we only analyzed 32 of the 46 study hospitals.

#### Numbers analysed

This is an intention to treat analysis. In each trial arm, there were 16 clusters (hospitals). We analyzed data from two samples: the consecutive (N = 658) and complicated samples (N = 209). Figure 1 shows the numbers of clusters and charts analysed in each trial arm.

#### Baseline data

Table 2 shows that the hospital and patient characteristics were very similar at the ALARM and control sites. There were, however, more district and regional sites among those not sampled; parity in patients from these hospitals was higher.

**Table 2:** Baseline characteristics of ALARM, control, and excluded sites

	ALARM sites*	Control sites*	**Not sampled**^**¥**^
Mali	6	7	9
Senegal	10	9	5
No. Capital sites	5	6	1
No. Regional/District	11	10	13
Mean patient age (SD)	25.7 (6.8)	25.8 (6.6)	25.2 (6.7)
Mean patient parity (SD)	2.3 (2.3)	2.1 (2.3)	3.3 (2.4)
Mean no. prenatal visits (SD)	3.1 (1.7)	3.0 (1.6)	2.8 (1.8)

^*^Calculated from the consecutive sample.

^**¥**^Data obtained from QUARITE trial database for same three-month study period (N = 5764 for age, 5776 for parity, and 5774 for prenatal visits).

Observations in the consecutive sample were well-distributed according to the intervention (48.5% at ALARM sites and 51.5% at control sites) and by location (33.4% in the capital versus 66.6% at district or regional hospitals). In the complicated sample, there were slightly more observations in the control arm than the ALARM arm (see Figure 1). When we centred the Complexity Index score around the mean, 45.4% of observations in the consecutive sample were below the mean while 54.6% were above it. In the complicated sample, 39.2% of observations were below the mean Complexity index score while 60.8% were above it. This is expected, as higher-level hospitals attract more cases of obstetrical complication.

### Outcomes and estimation

For the consecutive sample (N = 658 patients), the mean CBCA score was 66.30 (SD 13.46) and the mean Complexity index score was 68.27 (SD 10.21). For the complicated sample (N = 209 patients), the respective means were 69.11 (SD 12.42) and 68.54 (SD 9.36). For 27 (4.1%) women we could not calculate a CBCA score due to missing information in the consecutive sample. A slightly higher proportion (6.7%) could not be calculated for the complicated sample.

In both samples, unadjusted analyses indicate that there were significantly greater mean CBCA scores in women treated at ALARM hospitals compared to women treated at control hospitals. This difference was mostly attributable to greater patient scores for clinical examination at admission and post-partum monitoring scores (Table 3).

**Table 3:** Unadjusted patient CBCA sores according to sample and ALARM intervention

	**Consecutive sample**			**Complicated sample**		
	**N**	**ALARM (% )**	**Control (%)**	**N**	**ALARM (%)**	**Control (%)**
Initial interview	639	82.3%	81.1%	203	81.8%	81.4%
First clinical exam	657	86.4%*	80.5%	209	84.6%	80.7%
Laboratory exams	654	33.3%	31.7%	204	40.2%	33.6%
Delivery and birth	657	63.3%	62.8%	207	68.1%	71.0%
Postpartum monitoring	655	56.2%*	46.1%	208	63.0%*	49.8%
Total	631	68.2%*	64.5%	195	71.5%*	67.1%

*p-value for t-test ≤ 0.05.

We also looked at differences in the proportions of women receiving good quality care (greater than 70% criteria attainment) versus moderate to poor care (70% or fewer criteria attained). In the consecutive sample, significantly more women at ALARM sites received good quality care compared to women at control sites (44.1% versus 29.7%, p = 0.000). In the complicated sample, a similar difference between ALARM and control sites was observed (50.0% at ALARM sites versus 37.4% at control sites, p = 0.08).

For the subsections of the CBCA questionnaire pertaining to the direct complications of severe pre-eclampsia/ eclampsia and post-partum haemorrhage, there were no significant differences in total scores between arms of the QUARITE trial. Overall, criteria attainment for these sections of the questionnaire was very low (Tables 4 and 5). However, there were notable differences between trial arms for specific criteria within each section. While not statistically significant, for most criteria ALARM sites tended to have greater individual criterion attainment with the exception of criteria related to laboratory tests.

**Table 4:** Criterion attainment for women with severe pre-eclampsia/eclampsia according to the ALARM International Program

**Criterion**	**ALARM (n,%)**	**Control (n,%)**	**p-value**
	**N = 68**	**N = 70**	
Anticonvulsant administered			
Magnesium sulphate	26, 38.2%	20, 28.6%	
Valium	7, 10.3%	7, 10.0%	
None	35, 51.5%	43, 61.4%	0.455
Blood pressure recorded every four hours after birth			
Yes	15, 22.1%	13, 18.6%	
No	53, 77.9%	57, 81.4%	0.675
Urinary output measured at least once in 24 hours			
Yes	22, 32.4%	22, 31.4%	
No	46, 67.6%	48, 68.6%	1.000
Test of bleeding time			
Yes	5, 7.4%	10, 14.3%	
No	63, 92.6%	60, 85.7%	0.275
Test of coagulation rate			
Yes	5, 7.4%	9, 12.9%	
No	63, 92.6%	61, 87.1%	0.346
Platelet count			
Yes	22, 32.4%	17, 24.3%	
No	46, 67.6%	53, 75.7%	0.346
Albumin test			
Yes	18, 26.5%	18, 26.1%	
No	50, 73.5%	51, 73.9%	1.00
**Total severe (pre-) eclampsia score**	**25.2%**	**23.2%**	**0.621**

**Table 5:** Criterion attainment for women with post-partum haemorrhage according to the ALARM International Program

**Criterion**	**ALARM (n,%)**	**Control (n,%)**	**p-value**
	**N = 58**	**N = 64**	
Pulse and blood pressure every 15 minutes for 2 hours after diagnosis			
Yes	8, 13.8%	6, 9.4%	
No	50, 86.2%	58, 90.6%	0.57
Injection of oxytocin or egometrine			
Yes	18, 31.0%	9, 14.1%	
No	40, 69.0%	55, 85.9%	0.03
Intravenous oxytocin perfusion			
Yes	27, 46.6%	23, 35.9%	
No	31, 53.4%	41, 64.1%	0.27
How the placenta was expulsed			
Spontaneously	10, 17.5%	13, 21.3%	
Manually	43, 75.4%	35, 57.4%	
Not recorded	4, 7.0%	13, 21.3%	0.05
Test of bleeding time			
Yes	7, 12.3%	19, 29.7%	
No	50, 87.7%	45, 70.3%	0.04
Test of coagulation rate			
Yes	7, 12.3%	18, 28.1%	
No	50, 87.7%	46, 71.9%	0.06
Platelet count			
Yes	28, 49.1%	35, 54.7%	
No	29, 50.9%	29, 45.3%	0.48
**Total post-partum haemorrhage score**	**36.7%**	**34.9%**	**0.586**

#### Predictors of CBCA score

We used the larger consecutive sample to assess predictors of CBCA score. Table 6 presents results from the mixed models analysis. After adjusting for all relevant covariates, ALARM was significantly associated with CBCA score. Women treated at ALARM sites have, on average, 5 percentage points’ greater CBCA scores than those treated at control sites. Country predicted CBCA score better than any other variable; women treated at sites in Senegal have lower average CBCA scores than women treated in Mali. Complexity score was associated with better CBCA score in Mali, but not in Senegal. The number of patient prenatal visits was not significantly associated with CBCA score, but it was a potential confounder and thus, we preferred to retain it in the final model.

**Table 6:** Mixed-models analysis of predictors of CBCA score (N = 618)

**Variable**	**Estimate**	**95% Confidence interval**	**P-value**
Intercept	0.756	0.706 – 0.806	0.000
ALARM			
Intervention	0.052	0.003 – 0.102	
Control	-	-	0.040
Country			
Senegal	−0.184	^−^0.240 – ^-^0.128	
Mali	-	-	0.000
Centred complexity score	0.006	0.002 – 0.011	0.005
Prenatal visits			
3 or less	−0.012	^−^0.028 – 0.003	
4 or more	-	-	0.108
Country-Complexity interaction			
Senegal*Complexity	−0.005	^−^0.011 – 0.000	0.055
Mali*Complexity	-	-	

**Figure 2:**
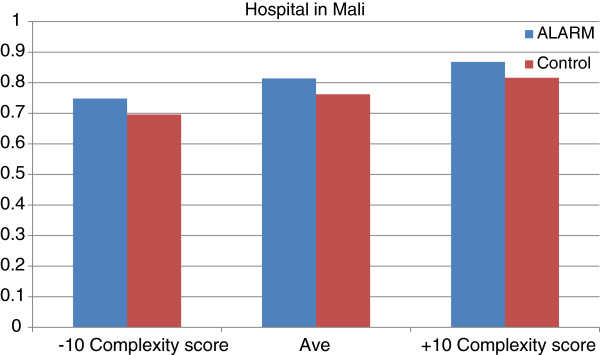
Relationship between CBCA and Complexity Index score according to ALARM in Mali.

**Figure 3:**
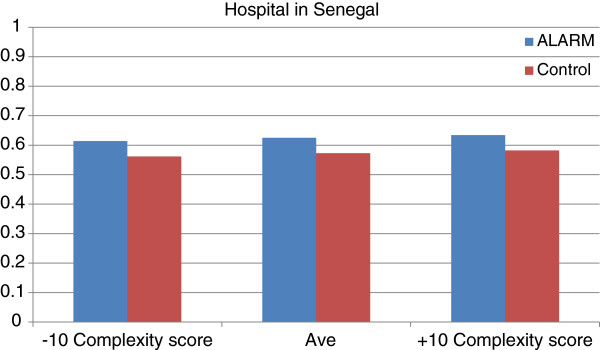
Relationship between CBCA and Complexity Index score according to ALARM in Senegal.

Figures 2 and 3 show relationship between ALARM and CBCA scores for three different scenarios of Complexity index score (10 points below the average score, the average score, and 10 points above the average score) for a woman with the recommended number of prenatal visits (4 or more).

In Mali, a ten-point increase above the average Complexity index score has a slightly more important effect on CBCA score than the ALARM intervention (6% improvement for Complexity index score versus 5% for ALARM). In Senegal, however, a 10-point increase above the average Complexity index score has a less important effect on CBCA score than ALARM (1% improvement for Complexity index score versus 5% for ALARM).

## Discussion

This was a sub-study of data from the QUARITE cluster randomized controlled trial. In this study, we attempted to parse out potential mechanisms of effect for the ALARM intervention. By doing so, we hoped to better understand how the intervention worked and what aspects of the intervention can be modified for future quality improvement efforts. Analyses from this study indicate that women treated at hospitals with the ALARM intervention have modestly greater mean CBCA scores. This suggests that the intervention is having the desired effect; it is associated with better obstetrical quality of care. Further, 15% more women are receiving good care (70% or greater criterion attainment) at ALARM hospitals when compared to control hospitals.

Results indicate that the intervention mostly improves clinical examination at admission and postpartum monitoring. There is also a trend suggesting that ALARM may be associated with increased use of magnesium sulphate in women with severe pre-eclampsia/ eclampsia. Finally, there was significantly greater administration of oxytocin for women with post-partum haemorrhage at ALARM sites and a greater proportion of women with post-partum haemorrhage at these sites were recorded as having had the placenta removed manually.

This study looks at the potential mechanisms of a complex quality improvement intervention and points to areas for future focus, specifically laboratory tests and birth and delivery care. It goes beyond simply assessing efficacy. By attempting to better elucidate what worked and why, we “look more closely inside the ‘black box’ of an intervention” ([25] p792), in order to better guide the development of future quality improvement programs. It takes a comprehensive look at quality of care evaluating both structure- (Complexity Index score) and process-level (CBCA score) components of care [26]. Quality of care is frequently proxied by the availability of hospital infrastructure and human resources based on the assumption that medical practice will improve as these resources increase [26]. We take a more nuanced view, acknowledging that context may determine the relative importance of each component of quality of care. In Senegal, for example, we saw that the ALARM intervention, which focused on medical practice, had more influence on intrapartum quality of care than resource availability. However, in Mali, where resources are lower than in Senegal, both the ALARM intervention and resource availability were important predictors of quality.

There are several strengths to this study. To our knowledge, this is the first validated CBCA to be used in a resource-limited setting to measure obstetrical care [21]. This was a very large audit of over 800 women (both samples) across 32 hospitals. Because of this, we were able to document the extensive variability in care that women received at referral facilities across the region. The breadth of the study, and the use of a validated CBCA instrument to quantitatively measure obstetrical care, gives us confidence that ALARM is, in fact, a promising quality improvement tool that can be applied in a wide variety of settings. In a trial of this size, one would expect effect dilution due to factors such as incomplete implementation of the program [27]. Given the inevitability of effect dilution, significant results showing quality of care improvements are encouraging and may suggest that the ALARM intervention can be generalized in low-income settings.

We sub-sampled women with direct complications that are both prevalent and have high case-fatality rates in West African hospital settings [28]. We were surprised to find that the ALARM intervention had a limited effect on the care of women with severe pre-eclampsia/ eclampsia. The care of this complication may be strongly influenced by resource availability. For example, in Mali, most district hospitals did not have sufficient laboratory resources to detect biological signs of severe pre-eclampsia, such as tests of proteinuria. These signs are frequently used as indicators for the use magnesium sulphate. However, even at sites with sufficient resources, complications appeared to go unrecognized. For both of the complications sampled, we were not able to rely exclusively on provider diagnoses. Sometimes, complications were simply not detected or euphemisms were employed (e.g. “endometriosis” following caesarean section instead of postpartum haemorrhage). As a result, we used multiple signs of complication (see participants section of methods) to detect cases severe pre-eclampsia/eclampsia and postpartum haemorrhage. By doing so, we increased the sensitivity of our case definition but may have reduced specificity (e.g. more false positives). Nonetheless, it is obvious that medical care for a complication cannot be improved if that complication is not acknowledged/ detected. Thus, future efforts need to improve the recognition of obstetrical complications.

There are limitations to this study. By not sampling all 46 hospitals in the QUARITE trial, we may have broke-down the initial randomization. For this reason, we considered potential confounders at both the hospital and patient levels. Given that hospitals in our sample were well-balanced by trial arm and by level (capital, regional, district) and that patient variables, such as age and parity, were not confounders, suggests that we did not introduce bias with the smaller sample. However, the proportion of district-level hospitals was greater in those sites not sampled (Table 2) and results from this study may be less generalizable to lower-level referral hospitals.

An additional limitation is related to the sample size for both of the direct obstetrical complications sampled. It is possible that we would have observed a significant result for the administration of magnesium sulphate at ALARM sites had we had a larger sample of clusters in both trial arms. There was very large variability at both the patient and hospital levels for the treatment of these complications. This reduced precision around the effect estimate and thus, our ability to detect a significant result. A larger sample would have allowed us to better interpret the laboratory results, as very few women received recommended laboratory tests for both complications. It should be noted that no patients in Mali were recorded as having tests of bleeding time or coagulation rate. Estimators for these criteria are thus very unstable.

Finally, the greatest weakness of CBCA is that it is dependent on what is recorded. In applying this tool, one assumes that what is recorded in the medical chart has actually been done and what has not been recorded, has not been done. This assumption may be flawed. For example, in Senegal, the name and qualification of the person who assisted the birth was recorded about a quarter of the time and in 80% of these cases, information was only recorded when the woman was seen by a medical doctor. Incomplete recording practices in Senegal may partially explain why CBCA scores were significantly lower there, compared to Mali. Nonetheless, charting is itself an indicator of quality of care. Good charting is necessary because the medical team following a woman may change over the course of a day and important decisions, such as when to conduct a caesarean section, are best made with the aid of tools such as the partograph [29].

## Conclusion

The ALARM intervention appears to improve certain aspects of the care process (clinical examination and post-partum monitoring), but other aspects (laboratory tests, delivery care) need further programmatic targeting. There is evidence suggesting that the program improves the care of women with post-partum haemorrhage, but that it is less effective for cases of severe pre-eclampsia/ eclampsia. Resource limitations, particularly in Mali, may be slightly more influential in determining the care of these patients (especially those with direct complications) than interventions targeting medical practice. Overall, these analyses demonstrate that future efforts in the region need to continue to target medical practice, but that a greater effect can be expected if resource availability is also improved.

## Abbreviations

CBCA: Criterion based clinical audit; MDR: Maternal death review; EmOC: Emergency obstetrical care.

## Competing interests

The authors declare that they have no competing interests.

## Authors' contributions

CP, AD, and MVZ were involved in the design, analysis, and writing of this manuscript. CP, MT, and AD were involved in data instrument development. CP and MT were involved in data collection. All authors revised and approved the final manuscript. All authors had full access to all of the data (including statistical reports and tables) in the study and can take responsibility for the integrity of the data and the accuracy of the data analysis.

## Authors' information

QUARITE trial research group

**Steering Committee:** Alexandre Dumont, Pierre Fournier, Michal Abrahamowicz, William Fraser, François Beaudoin, Slim Haddad.

**Data management:** Anna Koné, Drissa Sia, Papa Dambé.

**Data Security and Monitoring Committee:** José Villar (Chair), Christiane Welffens-Ekkra, Allan Donner.

**Participating institutions and Staff:** Society of Obstetricians and Gynaecologists of Canada (Ottawa, Canada): André Lalonde, François Beaudouin, Astrid Buccio, Jean-Richard Dortonne, François Couturier, Gilles Perrault, Pierre Levesques**;** CRCHUM (Montreal, Canada): Pierre Fournier, Slim Haddad, Anna Kone, Drissa Sia, Caroline Tourigny; Institut de recherche pour le développement (Dakar, Senegal): Alexandre Dumont, Mamadou Yatoudème Ndiaye, Papa Dambé; Centre de Recherche du CHU Sainte-Justine (Montreal, Canada): Sylvie Cossette, Carole Gariépy, François Beaudoin, William D. Fraser; CAREF (Bamako, Mali): Mouhamadou Gueye, Mamadou Kani Konate**;** HYGEA (Dakar, Sénégal): Idrissa Diop, Amadou Sow. **Participating hospitals in Senegal:** Centre Hospitalier Universitaire Abass Ndao: Prosper Bamboky, Keita Soma Diallo; Hôpital Principal: Pierre Dionne, Lô Asta; Centre Hospitalier Régional El Amadou Sahir Louga: Voulimata Fall, Maty Diop Guèye, Fatoumata Dedhiou; Centre Hospitalier Régional de Tambacounda: Maréme Soda Samba, Fatou Cissé Seck; Centre de Santé de Référence de Kédougou: Sène Doudou, Aïssatou Samba; Centre de Santé de Référence de Bakel: Yaya Baldé, Astou Guèye Thiombane; Centre de Santé de Référence de Goudiry: Julien Manga, Aïssata Sy Ndiaye, Absa Sene; Centre de Santé de Référence de Koungel: Mamadou Sarr, Diahaby Aminata Sylla; Centre Hospitalier Régional de Thiès: Fatou Rachel Sarr, Mbaye Khady Sarr; Centre Hospitalier Régional de Diourbel: Malick Gueye, Mbaye Binetou Diatta; Centre Hospitalier Matlaboul Fawzeïni: Mouhamadou M Seck, Niane Coumba Sarr Guéye; Centre Hospitalier Régional de Saint-Louis: Lamine Diouf, Oumou Kalsoum Fall; Centre de Santé de Référence de Richard Toll: El Hadji Lamine Dieye, Fall Ndèye Niang; Centre Hospitalier Départemental de Ourossogui: Charles Fall, Mame Laina Diattara, Astou Traoré; Centre Hospitalier Départemental de NDioum : Sidy Dieye, Ousmane Thiam, Kadiata Seck, Coumba Mbow, Aminata Dime; Centre Hospitalier Régional de Ziguinchor: Guy Boukar Faye, Badiane Aida Gaye; Centre de Santé de Référence de Sédhiou: Kalidou Konté, Fatou Ndoye; Centre Hospitalier Régional de Kolda: Aïdara Seynabou Sylla, Jacqueline F Ngom; Hôpital Général de Grand YOFF: Mariéme Fall, Aïssatou Diouf; Centre Hospitalier Régional El Hadji Ibrahima Niass: Dembo Girassy, Maye Seck Ly; Centre de Santé de Référence Nabil Choucaire: Seynabou Beye, Djipméra Fatou Ndiaye; Centre de Santé de Référence Youssou Mbargane: Dieynaba Ndao Ndiaye, Khady Fall; Centre de Santé de Référence Ndamatou Touba: Tacko Seck Leye ; Hôpital de Pikine: Codou Séne, Amsatou Cisse. **Participating hospitals in Mali:** Centre de Santé de Référence de Fana: Salif Sidibé, Aïssata Daba Traoré; Centre de Santé de Référence de Dioila: Broulaye Diarra, Korotoumou Doumbia; Centre de Santé de Référence de Markala: Bintou Coulibaly, Bintou Cissé; Hôpital Régional de Kayes: Mahamadou Diassana, Drissa Konaté, Dienfra Diarra; Centre de Santé de Référence de Niono: Moussa Modibo Diarra, Soundiè Fané; Hôpital Régional de Ségou: Haka Kokain, Souma Boiaré; Centre de Santé de Référence de San: Aliou Bagayogo, Diamilatou Diallo; Hôpital régional de Mopti: Famakan Kané, Pierre Coulibaly, Fatoumata Tolo, Fatoumata Dolo; Centre de Santé de Référence de Bougouni: Daouda Goïta, Bintou Sidibé; Centre de Santé de Référence de Koutiala: Moustapha Coulibaly, Sadio Tounkara, Mariam Beledogo; Hôpital régional de Sikasso: Mala Sylla, Cécile Dembélé, Rokia Touré; Centre de Santé de Référence de Kadiolo: Emilien Diarra, Ramata Fofana; Centre de Santé de Référence de Yanfolila: Aliou Coulibaly, Kadiatou Samaké; Centre de Santé de Référence Commune I de Bamako: Modibo Soumaré, Haoua Ba, Salimata Coulibaly; Centre de Santé de Référence Commune II de Bamako: Lassana Sissoko, Penda Fané, Hamsa Maïga; Centre de Santé de Référence Commune IV de Bamako: Moustapha Touré, Fanta Koné; Centre de Santé de Référence Commune V de Bamako: Oumar Soumana Traoré, Assitan Dembélé; Centre de Santé de Référence Commune VI de Bamako: Aminata Cissé, Feue Aminata Yattara, Katahit Zeneba; Centre Hospitalier Universitaire Point G de Bamako: Maïga Bouraïma,Kadiatou Traoré; Centre de Santé de Référence de Koulikoro: Hamadou Coulibaly, Oumou Konaté; Centre de Santé de Référence de Nioro du Sahel: Youssouf Coulibaly, Maïmouna Kassibo, Binta Kontao; Centre de Santé de Référence de Macina: Moussa Kanté, Diakité Diallo, Aminata Koné.

## Pre-publication history

The pre-publication history for this paper can be accessed here:

http://www.biomedcentral.com/1471-2393/13/24/prepub
